# Biomechanical analysis between Orthofix® external fixator and different K-wire configurations for pediatric supracondylar humerus fractures

**DOI:** 10.1186/s13018-018-0893-z

**Published:** 2018-07-28

**Authors:** Wen-Chao Li, Qing-Xu Meng, Rui-Jiang Xu, Gang Cai, Hui Chen, Hong-Juan Li

**Affiliations:** 1 Department of Pediatric Surgery, Chinese People’s Liberation Army General Hospital, Beijing, 100853 China; 2 Department of Basic Surgery, Affiliated Hospital of Hebei University, Baoding, 071002 China; 3 Department of Orthopaedic, Yu Huang Ding Hospital of Yantai, Yantai, 370600 China

**Keywords:** Supracondylar humerus fracture, Pediatric, Biomechanical stability, External fixation

## Abstract

**Background:**

Closed reduction and percutaneous fixation are considered as the optional treatments for displaced supracondylar humerus fractures. However, there was no published report about the biomechanical analysis in Orthofix® external fixator. In this study, we developed a model of supracondylar humerus fractures and compared the biomechanical analysis of external fixator and different K-wires configurations in order to evaluate the stability of external fixator in supracondylar humerus fractures.

**Methods:**

We developed an anatomic humerus model by third-generation synthetic composite, and 60 synthetic humeris were osteotomized to simulate the humeral transverse supracondylar fracture. Those fractures were reduced and fixed by external fixator or K-wires, and then biomechanical analysis was performed in extension, varus, valgus, and internal and external rotation loading. A paired-sample *t* test was used to evaluate the distance at the fracture site between the external fixator and K-wire configurations.

**Results:**

During all direction loading, there was a significant statistical difference between external fixator and K-wires (*P* < 0.001 for all pairwise comparisons). In extension and internal rotation loading, the external fixator and three crossed K-wires had no comparable stiffness values (*P* = 0.572; *P* = 0.795), and both were significantly greater than two crossed and lateral K-wires (*P* < 0.05). In external rotation loading, there was no significance between the external fixator and K-wire configurations except two lateral K-wires (*P* > 0.05). In valgus loading, the stability of the external fixator was less than that of three crossed K-wires (*P* = 0.001) but was not significantly different with those of two crossed or three lateral K-wires (*P* = 0.126; *P* = 0.564). In varus loading, the stability of the external fixator was larger than those of two and three lateral K-wires (*P* = 0.000; *P* = 007).

**Conclusions:**

External fixator could provide enough stability for pediatric supracondylar humerus fractures without the injury of the ulnar nerve. Besides, it could enhance the rotational stiffness of the construct in rotation loading to avoid the complication of cubitus varus.

## Background

Supracondylar fracture of the distal humerus is a common fracture in the pediatric population, accounting for approximately 60% of all fractures of the elbow [1]. Since1948, Swenson firstly described two K-wires of different sizes for closed reduction of supracondylar humerus fractures [2]. The classical treatment of displaced supracondylar humeral fractures is closed reduction and percutaneous fixation of Kirschner wires (K-wires). Previous studies have shown that medial and lateral crossed-pin fixation provided more stability in biomechanical analysis than two lateral pin fixation [3]. However, crossed K-wire placement is associated with the risk of iatrogenic ulnar nerve injury up to 3 to 4%. Lee et al. [4] reported that three lateral divergent or parallel pin fixations were effective and safe in avoiding iatrogenic ulnar nerve injury in supracondylar humeral fractures. In Bogdan et al.’s [5] study, the humero-ulnar external fixation is a good alternative to lateral or crossed pinning in supracondylar humeral fractures. The optional K-wire configuration could provide the adequate stability of fracture without the risk of neurovascular injury.

The biomechanical analysis in different configurations, including two cross, two divergent lateral, three divergent lateral, a medial and two lateral, and external fixator with radially K-wire, were performed in supracondylar humerus fractures [6–9]. In our department, we performed the closed reduction and percutaneous fixation of the Orthofix® external fixator in the treatment for supracondylar humerus fractures with successful clinical outcomes. However, there have been no published reports of biomechanical analysis in Orthofix® external fixator in supracondylar humerus fractures. In this study, we developed the model of supracondylar humerus fractures and compared the biomechanical analysis between the external fixator and K-wire configurations in order to evaluate the stability of the external fixator in supracondylar humerus fractures.

## Methods

### Specimen preparation

The anatomic humerus model was developed using a third-generation synthetic composite for this study (Sawbones #3404; Pacific Research Laboratories, Vashon Island, WA) (Model #1028; Pacific Research Laboratories Inc., Vashon, WA, USA). Sixty synthetic humeri were osteotomized at the level of the coronoid and olecranon fossae to simulate a humeral transverse supracondylar fracture. A 10° oblique osteotomy was created with a standardized jig starting at the proximal edge of the olecranon fossa descending to the coronoid fossa.

**Fig. 1:**
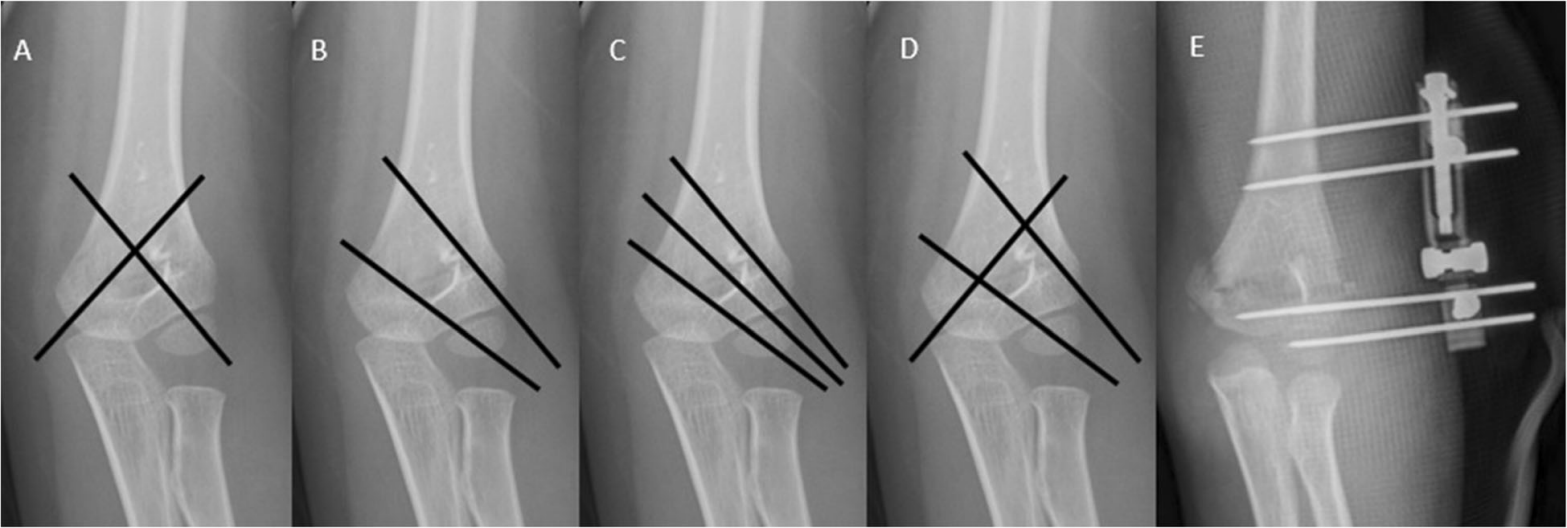
Schematic of the four K-wire configurations and the external fixator. **a** Two crossed K-wires. **b** Two lateral divergent K-wires. **c** Three lateral divergent K-wires. **d** A medial and two lateral divergent K-wires (three crossed K-wires). **e** External fixator

After the reduction of fracture fragment, the fracture was then fixed by stainless steel K-wires. We used 1.5-mm K-wires for children with weight less than 15 kg and 2-mm K-wires for other children. Prof Wen-Chao Li had performed the surgery of fracture reduction by radiography-guided fluoroscopy. The external fixator and four different K-wire configurations were used for stabilizing the supracondylar humerus fracture (Fig. 1).

I. Two crossed K-wires [2]: a medial and a lateral K-wire.
II. Two lateral divergent K-wires [10]: a lateral pin was placed parallel to the lateral metaphyseal flare of the humerus. The second pin crossed the osteotomy site at the medial edge of the coronoid fossa.
III. Three lateral divergent K-wires [9]: two lateral divergent K-wires combined with a K-wire placed between the two divergent pins.
IV. A medial and two lateral divergent K-wires (three crossed K-wires) [7]: two lateral divergent pins combined with a medial K-wire.
V. External fixator: the first screw was inserted into the osteoepiphysis of capitulum humeri parallel to the articular surface of the distal humeral. The second screw in the distal fracture was parallel to the first screw. Both screws pass through the middle metaphysis of the distal end of the humerus and were fixed to the external fixing frame. The others screws were inserted in the proximal fracture. Those screws was fixed in the predesigned position of external fixator. The size of screw in the external fixator is 2 mm.

The specimens with reduction fracture by different fixations were fluoroscopically imaged to ensure consistent orientation between K-wires. Each group including 12 specimens of different fixations were tested in biomechanical analysis by MTS 858 MiniBionix materials testing machine (MTS, Eden Prairie, MN, USA). The testing sequence of five fixations in each group was varied to minimize sequencing effects. We performed the mechanical tests in each fixation to evaluate the different directions of loading: flexion, extension, varus, valgus, internal, and external rotation.

The proximal end of each humerus was embedded with a commercially available two-part epoxy resin and placed into a custom testing machine to provide secure fixation of the specimen during testing. The distal fragment was placed in a custom mold to allow free motion through the fracture site during testing without influencing the position of the K-wires. In the test of extension, varus, and valgus, the load was applied to and measured from the distal fragment at a quasi-static displacement rate of 0.5 mm/s to a maximum of 5 mm. Load (N) and displacement (mm) were measured at the distal fragment and recorded at 10 Hz using an MTS Sintech 1/G material testing machine (MTS Corporation). In the test of internal and external rotations, the custom molds at the distal fragment of the fracture were clamped with two flat plates. Torsion was applied at a quasi-static angular displacement rate of 0.5°/s to an end point of ± 10° using an MTS 858 Minibionix (MTS Corporation, Eden Prairie, MN). Torque (Nm) and angular rotation (mm) were recorded at 10 Hz.

### Statistical analysis

Analysis of variance with repeated measurements was used to evaluate differences in the stiffness values of the external fixator and different K-wire configurations. A *P* value of 0.05 or less was considered statistically significant. Multiple *t* test comparisons were performed to determine differences among the separate fixation when significance was found. A paired-sample *t* test was used to evaluate differences in the distance between the different fixations at the fracture site for the external fixator and K-wire configurations.

## Results

During the test of all the loading conditions, there were no instances of permanent displacement of the fracture resulting in loss of fixation or pin deformation. During the extension loading, there was a significant statistical difference between K-wires and the external fixator (*P* = 0.000 for all pairwise comparison). The external fixator and three crossed K-wires had no comparable stiffness values (average ± standard deviation, 7.5 ± 1.4 and 7.9 ± 1.7 N/mm, respectively), and both were significantly greater than two crossed and lateral K-wires. Two lateral K-wires were significantly smaller than other groups (*P* < 0.05). Two crossed with stiffness value of 6.2 ± 1.0 N/mm was larger than two lateral crossed, but smaller than three crossed K-wires and external fixator (Table 1).

**Table 1:** Construct stiffness data in extension loading direction for different K-wire configurations or external fixator

Extension loading	Magnitudes (N/mm)	ANOVA (*P*)	Comparison (*P*)				
	Mean ± SD^†^		(I)	(II)	(III)	(IV)	(V)
Two crossed K-wires (I)	6.2 ± 1.0	*0.000*	–	*0.048*	0.605	*0.028*	*0.013*
Two lateral K-wires (II)	5.1 ± 1.3		–	–	*0.038*	*0.000*	*0.000*
Three lateral K-wires (III)	6.5 ± 1.5		–	–	–	0.140	0.066
Three crossed K-wires (IV)	7.5 ± 1.4		–	–	–	–	0.572
External fixator (V)	7.9 ± 1.7		–	–	–	–	–

Italicized values are significantly different between two groups (*P* < 0.05)

^†^The values are given as mean ± standard deviation

The maximal loads and torques required to produce the prescribed displacement and rotation exhibited similar trends to the stiffness results. During the varus and valgus loading, there was significant statistical difference between all K-wires of configurations and external fixator (*P* < 0.001). The stiffness of three crossed K-wires (21.2 ± 3.1 Nm/mm; 17.9 ± 2.3 Nm/mm) was larger than that of two lateral K-wires (14.1 ± 2.0 Nm/mm, *P* < 0.001; 13.5 ± 1.7 Nm/mm, *P* < 0.001) and three lateral K-wires (15.4 ± 2.3 Nm/mm, *P* < 0.001; 15.8 ± 2.0 Nm/mm, *P* = 0.042), and there was no significant difference between three and two crossed K-wires (20.4 ± 2.4 Nm/mm, *P* = 0.526; 16.7 ± 2.1 Nm/mm, *P* = 0.238). In varus loading, there was no significant difference between the stiffness of the external fixator (18.9 ± 2.9 Nm/mm) and three crossed K-wires (21.2 ± 3.1 Nm/mm, *P* = 0.015) or two crossed K-wires (20.4 ± 2.4 Nm/mm, *P* = 0.042). But the stiffness of the external fixator was larger than that of two lateral K-wires (14.1 ± 2.0 Nm/mm, *P* = 0.000) and three lateral K-wires (15.4 ± 2.3 Nm/mm, *P* = 0.007). In valgus loading, the stiffness of the external fixator (15.3 ± 1.8 Nm/mm) was less than that of three crossed K-wires (17.9 ± 2.3 Nm/mm, *P* = 0.001) (Table 2).

**Table 2:** Construct stiffness data in varus and valgus loading directions for different K-wire configurations or external fixator

Varus loading	Magnitudes (Nm/mm)	ANOVA (*P*)	Comparison (*P*)				
	Mean ± SD^†^		(I)	(II)	(III)	(IV)	(V)
Two crossed K-wires (I)	20.4 ± 2.4	*0.000*	–	*0.000*	*0.000*	0.526	0.224
Two lateral K-wires (II)	14.1 ± 2.0		–	–	0.194	*0.000*	*0.000*
Three lateral K-wires (III)	15.4 ± 2.3		–	–	–	*0.000*	*0.007*
Three crossed K-wires (IV)	21.2 ± 3.1		–	–	–	–	0.104
External fixator (V)	18.9 ± 2.9		–	–	–	–	–
Valgus loading	Magnitudes (Nm/mm)	ANOVA (*P*)	Comparison (*P*)				
	Mean ± SD^†^		(I)	(II)	(III)	(IV)	(V)
Two crossed K-wires (I)	16.7 ± 2.1	*0.000*	–	*0.000*	0.339	0.238	0.126
Two lateral K-wires (II)	13.5 ± 1.7		–	–	*0.012*	*0.000*	*0.033*
Three lateral K-wires (III)	15.8 ± 2.0		–	–	–	*0.042*	0.564
Three crossed K-wires (IV)	17.9 ± 2.3		–	–	–	–	*0.001*
External fixator (V)	15.3 ± 1.8		–	–	–	–	–

Italicized values are significantly different between two groups (*P* < 0.05)

^†^The values are given as mean ± standard deviation

During the internal and external rotation loading, there were significant statistical differences between all K-wires of configurations and the external fixator (*P* < 0.001). There was no significant difference between three crossed K-wires and the external fixator (*P* > 0.05). In internal rotation loading, the stiffness in both fixations (117 ± 18 Nmm/degree; 119 ± 16 Nmm/degree) was larger than those of two and three crossed K-wires and three lateral K-wires (*P* < 0.05). In external rotation loading, the stiffness in both fixations (121 ± 16 Nmm/degree; 120 ± 19 Nmm/degree) was not significantly different with two crossed and three lateral K-wires (*P* > 0.05), but larger than that of two lateral K-wires (*P* < 0.001). During all the five loading conditions, there was a trend for two crossed K-wires to have a greater stiffness value than two lateral K-wires (*P* < 0.05), and three lateral K-wires had a similar statistically significant difference except for varus loading (*P* = 0.194) (Table 3).

**Table 3:** Construct stiffness data in internal rotation and external rotation loading direction for different K-wire configurations or external fixator

Internal rotation loading	Magnitudes (Nmm/degree)	ANOVA (*P*)	Comparison (*P*)				
	Mean ± SD^†^		(I)	(II)	(III)	(IV)	(V)
Two crossed K-wires (I)	101 ± 16	*0.000*	–	*0.024*	0.769	*0.039*	*0.021*
Two lateral K-wires (II)	84 ± 15		–	–	*0.032*	*0.000*	*0.000*
Three lateral K-wires(III)	99 ± 14		–	–	–	*0.022*	*0.008*
Three crossed K-wires (IV)	117 ± 18		–	–	–	–	0.795
External fixator (V)	119 ± 16		–	–	–	–	–
External rotation loading	Magnitudes (Nmm/degree)	ANOVA (*P*)	Comparison (*P*)				
	Mean ± SD^†^		(I)	(II)	(III)	(IV)	(V)
Two crossed K-wires (I)	108 ± 15	*0.000*	–	*0.032*	0.451	0.077	0.134
Two lateral K-wires (II)	93 ± 14		–	–	*0.005*	*0.000*	*0.000*
Three lateral K-wires (III)	102 ± 14		–	–	–	0.249	0.432
Three crossed K-wires (IV)	121 ± 16		–	–	–	–	0.900
External fixator (V)	120 ± 19		–	–	–	–	–

Italicized values are significantly different between two groups (*P* < 0.05)

^†^The values are given as mean ± standard deviation

## Discussion

Supracondylar humeral fractures are common in children and account for 13~ 16% of all pediatric fractures. The goals of the treatment for displaced supracondylar humerus fracture are closed or open anatomical reduction and maintaining the reduction of fracture without iatrogenic nerve injury [11]. Those previous studies have reported biomechanical analysis with different K-wire configurations in supracondylar humeral fractures. However, there have been no published reports of biomechanical analysis in the external fixator in supracondylar humerus fractures. In our study, we developed a model of supracondylar humerus fractures and compared biomechanical analysis in the external fixator with different K-wire configurations to evaluate the stability of the external fixator in the humerus fracture. In this study, there was no significant difference between the external fixator and three crossed K-wires in extension, rotation, and varus loading (*P* > 0.05), and the stability of the external fixator was less than that of three crossed K-wires in valgus loading (*P* = 0.001). Besides, the external fixator provided more stability than two crossed K-wires in extension (*P* = 0.013) and internal rotation (*P* = 0.021) loading and was not significantly different than two crossed K-wires in other direction loading (*P* > 0.05).

It is well known that two crossed K-wires could provide anatomical reduction and the stability of fixation to lower the incidence of Volkmann ischemia with the elbow less than 90°. However, ulnar nerve injury is a common complication when the K-wire is inserted in the medial direction. Those previous studies reported that the frequency of iatrogenic ulnar nerve injuries by the medial placement of K-wires ranges from 1.4 to 15.6% [12]. Babal et al. [13] concluded that the medial pin carried the greater overall risk of nerve injury as compared with the lateral pin-only construct and that the ulnar nerve was at risk of injury in patients who had medial pins. Brauer et al. [14] reported the probability of iatrogenic nerve injury was 1.84 times higher with medial and lateral pins than that with lateral entry pin. In extension loading, Feng et al. reported that the two lateral divergent pins were less stable than the two crossed pins [9], which was similar with the result of our study (*P* < 0.05). The external fixator and three crossed K-wires had no comparable stiffness values (7.5 ± 1.4 N/mm; 7.9 ± 1.7 N/mm, respectively), and both were significantly greater than two crossed and lateral K-wires. The external fixator could be regulated in extension direction by a spanner. Besides, the external fixator could provide the stability of the distal and proximal fracture by those screws.

Configurations using lateral-only entry K-wires have been recommended to decrease the risk of iatrogenic injury to the ulnar nerve. Zionts et al. [15] compared the stability provided by different pin configurations and demonstrated that crossed-pin configuration provides the most stable torsional fixation, followed by the fixation achieved with two and three lateral pins. In our study, two crossed K-wire configuration was significantly stiffer than two lateral divergent K-wires, especially in varus and valgus direction loading. Adding a third lateral K-wire to the crossed or two lateral K-wire configuration could provide more stability in the fracture than previous K-wires, but the difference was not significant in the same directions. This finding suggests that the surgeon faced with a biomechanically unstable fracture pattern or a less-than-anatomic reduction may use additional lateral K-wires to supplement biomechanical stability. In Larson et al.’s study [16], the three crossed-pin construct was most stable in the fracture followed by three lateral pins, and two lateral divergent pins demonstrated the least torsional stability. However, Srikumaran et al. [8] reported that gross observation suggests that the addition of a third lateral pin to the crossed configuration increased cortical destruction, making the construct less stable in extension. In valgus loading, the stiffness of the external fixator (15.3 ± 1.8 Nm/mm) was less than that of the three crossed K-wires (17.9 ± 2.3 Nm/mm, *P* = 0.001). The screws in the external fixator with good elasticity were likely to bend as a result of interval of fracture fragment during the external loading. Therefore, the stability of the external fixator was less than that of the three crossed K-wires. However, during varus loading, the internal of fracture fragments was compressed without displacement, and the screws were kept to maintain the stability of the fracture fragment. Therefore, there was no significant difference between the stiffness of the external fixator (18.9 ± 2.9 Nm/mm) and the three crossed K-wires (21.2 ± 3.1 Nm/mm, *P* = 0.015).

Mechanical rotation stability of the different fixations in supracondylar humeral fracture is a major factor to avoid the development of cubitus varus. Cubitus varus has been considered as being just a cosmetic problem by many authors. The ulnar insertion of an anti-rotation wire into the distal fragment reinforces the stability if internal rotation loading is applied and stabilizes the ulnar column of the distal humerus [17]. Wang et al. [18] reported that there was no statistical difference between the two medial pins and the two crossed-pin configurations (*P* = 0.06 and 0.75, respectively) in internal and external rotation testing, but they were significantly greater than two lateral pins (*P* = 0.003 and 0.004; *P* = 0.001 and 0.02, respectively). In our study, the crossed K-wires provided more stability than two lateral K-wires (*P* = 0.024; *P* = 0.032), which was similar with the previous study. Besides, the stiffness of two lateral K-wires (84 ± 15 Nmm/degree; 93 ± 14 Nmm/degree) was less than those of the external fixator and other three K-wire configurations (*P* < 0.05). The external fixator could provide more stability and resistance to internal rotation than two and three crossed K-wires (*P* = 0.021; *P* = 0.008), while in external rotation loading, the external fixator was not statistically significantly different with two and three crossed K-wires (*P* = 0.134; *P* = 0.432).

In Feng et al.’s study [9], two medial divergent pins and two crossed pins had comparable stiffness values and both were significantly greater than two lateral pins during the rotation loading, which was similar with our study (*P* = 0.024; *P* = 0.032). Previous biomechanical studies have shown that crossed pins provide greater rotational stability than both parallel lateral and divergent lateral pin constructs for transverse fractures [10, 19]. Bloom et al. [20] reported that the addition of the third K-wire compared with an anatomically reduced two crossed K-wire configuration resulted in increased stiffness of the model for all loading directions. Besides, the external fixator could provide similar resistance to internal and external rotation loading with three crossed K-wires. The above biomechanical analysis showed that the external fixator could provide enough rotation loads and torques in supracondylar humeral fractures.

In this article, we evaluated the biomechanical analysis in the external fixator and K-wire configuration for the displaced supracondylar humerus fractures. However, this study has significant limitations. In our study, we performed the synthesis models to evaluate the biomechanical analysis of fracture reduction techniques, but the transverse osteotomy of the humeral model could not account for the variability of fracture line in clinical fracture. The next study should be performed in a coronal medial obliquity and lateral obliquity model. The surrounding of the fracture including the muscle, periosteum, vessel, and nerve could contribute to the choice of fracture and fragment stability. The model in our study without structural variation or inconsistency was desirable as it mainly involves relative comparisons of stiffness. In experiment study, we could evaluate the biomechanical analysis in the single direction loading in the synthesis model. However, the physiological loading in clinical usually contain two or three directions loading. The complex direction loading in the fracture model can be performed by computer-assisted analysis. The external fixator and different K-wire configurations is performed to evaluate the biomechanical analysis to select the proper fixation.

## Conclusions

The external fixator could provide enough stability without the injury of the ulnar nerve in treatment of a supracondylar humeral fracture to reduce the displacement of the fragments. Besides, it enhances the rotational stiffness of the construct in rotation loading to avoid the complication of cubitus varus. The stability of the external fixator was less than that of three crossed K-wires in valgus loading, but there was no significant difference between the external fixator and two crossed K-wires. Long-term results of this new variation of the external fixator will be evaluated in a clinical setting.

## Abbreviation

K-wire: Kirschner wire
